# Authors` response to “Efficacy of noninvasive ventilation in patients with COVID-19”

**DOI:** 10.1186/s13054-022-04267-8

**Published:** 2022-12-22

**Authors:** Kamil Polok, Jakub Fronczek, Wojciech Szczeklik

**Affiliations:** Center for Intensive Care and Perioperative Medicine, Jagiellonian University Medical College, Ul. Wrocławska 1-3, 30 – 901, Kraków, Poland

We thank Shen and colleagues for their interest in our paper on the use of noninvasive ventilation in older adults with COVID-19 [1].

We believe that the relatively high NIV failure rate in our study is multifactorial. First, participants in our study were significantly older than the sample enrolled by Arabi and colleagues (76 vs 58 years) in a study cited by the authors [2]. Despite not having collected granular data on pre-ICU respiratory support, we agree that many patients with severe COVID-19-related respiratory failure received high-flow nasal oxygen therapy or NIV outside of ICUs, potentially leading to a delay of intubation and worse outcomes.

We remain cautious about judging the appropriateness of NIV administration in the setting of an extraordinary disruption of standard treatment pathways during the studied period. Due to a shortage of ICU beds, the propensity to limit life-sustaining (LST) treatment was likely higher than before the pandemic [3, 4]. Nevertheless, we are confident that some patients in the primary NIV group would have been intubated in normal circumstances.

Comparison between the primary NIV and primary IMV group suggested a higher 30-day mortality rate in the former group. This observation was confirmed in a sensitivity analysis excluding patients in whom LST was limited when primary respiratory support modality was used. Conversely, another sensitivity analysis excluding all patients with LST limitation showed no difference between the groups. It might suggest that this difference is driven by high mortality in patients primarily treated with NIV who were subsequently intubated. This is in-line with our analysis showing an association between pre-intubation NIV duration and 30-day mortality. Nevertheless, we agree that further studies are needed to establish optimal respiratory support modes in different patient populations of critically ill patients.

## Data Availability

Not applicable.
